# Stramonium Cigars

**Published:** 1846-12

**Authors:** 


*Stramonium Cigars.*—Stramonium has for a long time been recommended as a remedy for asthma and other disorders of the chest, and it is frequently used in a pipe with a portion of tobacco. Mr. Butler, of Covent Garden, has recently introduced cigars composed of the leaves of this plant, and we have been informed by several patients who have tried them, that the plan is more convenient than the one formerly adopted, and that the remedy is quite as efficacious, if not more so. A short time ago, a caution was issued to chemists, to the effect that these cigars could not legally be sold without a cigar license, and on this account we have deferred our notice of them. But we are now informed that this objection is overruled, it being decided that the stramonium cigars may be sold without a license as a remedy, as no tobacco enters into their composition.

In France, not only stramonium but several other medicinal plants are used in the form of cigars; and we have been informed that advantage has been found to result from the practice in some cases.—*Pharm. Journal.—Dublin Medical Press.—Ibid.*

